# Effects of carvedilol treatment on cardiac cAMP response element binding protein expression and phosphorylation in acute coxsackievirus B3-induced myocarditis

**DOI:** 10.1186/1471-2261-13-100

**Published:** 2013-11-14

**Authors:** Ge Li-Sha, Chen Yi-He, Zhou Na-Dan, Zhang Teng, Li Yue-Chun

**Affiliations:** 1 Department of Pediatric, Second Affiliated Hospital of Wenzhou Medical College, Wenzhou 325000, China; 2 Department of Cardiology, Second Affiliated Hospital of Wenzhou Medical College, Wenzhou 325000, China

**Keywords:** Viral myocarditis, cAMP response element binding protein, Carvedilol

## Abstract

**Background:**

The role of β-adrenergic stimulation on viral myocarditis has been investigated in animal models of viral myocarditis. Excess stimulation of β-adrenergic receptors by catecholamines causes phosphorylation/activation of cAMP response element binding protein (CREB) by the cAMP signaling pathway. CREB as an important regulator of gene expression mediates the cardiovascular remodeling process and promotes anti-inflammatory immune responses. However, the CREB expression and phosphorylation have not been studied, and the effects of carvedilol (a nonselective β-adrenoceptor antagonist) on the CREB has not been investigated in the setting of acute viral myocarditis.

**Methods:**

This study was therefore designed to examine the effects of carvedilol on the transcriptional factor CREB in a murine model of acute viral myocarditis. In a coxsackievirus B3 murine myocarditis model (Balb/c), effects of carvedilol on plasma noradrenaline, heart rate and blood pressure, myocardial histopathological changes and fibrosis, cardiomyocyte apoptosis, cardiac CREB and phosphorylated CREB, cytokine levels, and viral RNA were studied.

**Results:**

The expression and phosphorylation of CREB were decreased with concomitant increase of IL-6 and TNF-α in murine coxsackievirus-induced acute viral myocarditis. The levels of IL-6 and TNF-α were correlated with the expression of CREB or phosphorylated CREB. Carvedilol increased the cardiac CREB expression and phosphorylation and decreased the plasma catecholamine levels and the production of IL-6 and TNF-α with amelioration of acute viral myocarditis.

**Conclusion:**

These results show that CREB may be involved in the pathophysiology of viral myocarditis and carvedilol exerts some of its beneficial effects by increasing the CREB expression and phosphorylation.

## Background

Viral myocarditis is a frequent cause of cardiac failure, especially in young adults [1]. Acute inflammation of the heart appears to be a major cause of sudden, unexpected death in persons younger than 40 years old [2] and often follows viral infection [3]. Enteroviruses, particularly the coxsackievirus B3 (CVB3), have been identified as the predominant cause of viral myocarditis in both animal models and humans [4]. Despite the well-characterized molecular structure of coxsackieviruses [5] and the successful use of common [6] or novel immunization procedures [7-9] in animal models, immune suppression trials [10] to reverse underlying active myocardial inflammation have been largely disappointing, and no specific treatment for viral myocarditis are in clinical use today. Therefore, elucidation of the fundamental mechanisms involved in the development of myocarditis, and the progression from virus infection to heart failure is important.

In patients with heart failure, plasma epinephrine and norepinephrine levels were elevated. Excess stimulation of the β-adrenergic receptor by catecholamines may contribute to the pathogenesis of congestive heart failure of various causes [11]. The cardiac responsiveness to β-adrenergic stimulation in heart failure is associated with gene expression alterations in myocardial regulatory proteins which are involved in the cAMP-dependent signal transduction [12-15]. The transcriptional control by the cAMP-response element binding protein (CREB) is a major mechanism regulating the gene expression by the cAMP-dependent signaling pathway [16-20]. CREB plays an important role in the regulation of a number of genes involved in the maintenance of normal cellular function [16-20]. Several studies suggested CREB as an important regulator of gene expression involved in the pathophysiology of heart failure [21-24]. Transgenic mice with cardiac myocyte-specific expression of dominant negative CREB showed impaired contractile response to isoproterenol stimulation as well as dilatation of the 4 chambers of the heart, mimicking idiopathic dilated cardiomyopathy [21]. Moreover, evidence from an increasing number of studies has shown that CREB mediates the cardiovascular remodeling process [25], including inflammation [26], cell migration [27], and apoptosis [28,29]. However, CREB in viral myocarditis has not been studied. The role of β-adrenergic stimulation on viral myocarditis has also been investigated in animal models of viral myocarditis. We and other investigators have recently reported that the circulating plasma levels of catecholamines were significantly increased in murine viral myocarditis [30,31]. These results implicated that the transcriptional factor CREB may be also involved in the pathophysiology of viral myocarditis. Therefore, in the present study, we examined the role of the transcriptional factor CREB in murine coxsackievirus-induced acute viral myocarditis.

Carvedilol, a third-generation, nonselective β-adrenoceptor antagonist that also possesses α_1_-adrenergic blocking, antioxidant, antiapoptotic, anti-inflammatory, and antifibrotic properties, has been shown to provide greater benefit than traditional β-adrenoceptor antagonists in chronic heart failure [32]. Recently, our and other studies have demonstrated protective effects of carvedilol in viral myocarditis [30,31,33-35]. The beneficial action of carvedilol in murine viral myocarditis may be at least partly due to its effects of catecholamine reduction [30,31,33-35]. In patients with heart failure, the reducion of catecholamines by β-blockade “restored” the changes of the cardiac regulatory proteins which are involved in the cAMP-dependent signal transduction [32,36,37]. Therefore, β-adrenoceptor antagonists may also be useful to “normalize” the altered CREB expression in viral myocarditis. However, its effects on the CREB in experimental viral myocarditis has not yet been investigated. Thus, in the present study, we studied the effects of carvedilol on the transcriptional factor CREB in a murine model of acute viral myocarditis induced by CVB3.

## Methods

### Ethics statement

The investigation conformed with the Guide for the Care and Use of Laboratory Animals published by the US National Institutes of Health (NIH Publication, 8th Edition, 2011), and all experiments were carried out in accordance with China Animal Welfare Legislation and were approved by the Wenzhou Medical College Committee on Ethics in the Care and Use of Laboratory Animals. For the survival study, individual mice were monitored daily and were euthanized when they displayed signs of myocarditis-associated morbidity such as excessive weakness and lethargy. All animals were anaesthetized with pentobarbital (100 mg/kg, one dose intraperitoneally) prior to sacrifice. Efficient anaesthesia was monitored through pinching the hind paw, when sufficiently sedated the mice were euthanized through cervical dislocation.

### Mouse model of viral myocarditis

Specific pathogen-free inbred, 4-week-old, male Balb/c mice, obtained from Shanghai Laboratory Animal Center, China, were inoculated intraperitoneally with 1.0 × 10^6^ plaque-forming units (pfu) of CVB3 (strain Nancy, ATCC VR-30) diluted in phosphate-buffered saline (PBS) to a final volume of 0.1 ml (n = 60). Group control was inoculated intraperitoneally 0.1 ml with normal saline solution (n = 30). The day of virus inoculation was defined as day 0.

### Drug administration

Carvedilol was obtained from Roche China Co. (Shanghai, China). Starting 24 h after infection carvedilol (10 mg/kg per day, n = 30) were administered by gavage for 14 consecutive days, whereas normal control group (n = 30) and myocarditis group mice (n = 30) received the normal saline solution in the same way. Eight surviving mice from each group were killed on day 7 or 14.

### Plasma noradrenaline

Plasma noradrenaline was measured using high-performance liquid chromatography and electrochemical detection. After mice were anesthetized intraperitoneally with pentobarbital (50 mg/kg), the arterial blood samples were taken and centrifuged at 3000 g for 15 min. The plasma were stored at −80°C for subsequent determination of noradrenaline concentration. The experiment was repeated 6 times for each sample.

### Hemodynamic measurements

HR and blood pressure (BP) were measured using a photoelectric tail cuff detection system (softron BP-98A from Japan) on conscious mice that had been pre-warmed for 10 minutes at 37°C in a thermostatically controlled heating cabinet. The values were averaged from at least three consecutive readings on each occasion.

### Survival rate

Survival was measured over a 14-day period.

### Myocardial histopathology

The ratio of heart weight to body weight (HW/BW) was calculated. The heart tissue was fixed in 10% formalin, embedded in paraffin, sectioned, and stained with hematoxylin and eosin. Several sections of each heart were scored blindly by two observers. The scores assigned to these specific sections were averaged. The extent of cellular infiltration and myocardial necrosis was graded and scored as follows: 0 = no lesion; 1 + = lesions involving <25% of the myocardium; 2 + = lesions involving 25% to 50%; 3 + = lesions involving 50 to 75%; and 4 + = lesions involving 75% to 100%.

### Masson trichrome staining

The heart tissue specimen was cut in a 5-mm-thick, fixed in 10% formalin, dehydrated and embedded in paraffin. The slices were stained with Masson’s trichrome stain (GENMED SCIENTIFICS INC. USA). Five visual fields were randomly selected in each slice stained with Masson’s trichrome under a microscope and measured by Image Pro Plus image analysis software. Collagen volume fraction (CVF) obtained from the average ratio of collagen area to total tissue area was used to evaluate the extent of interstitial fibrosis. CVF excluded scars and perivascular collagen areas.

### Detection of apoptosis

Apoptosis was detected in myocardial tissue sections using the terminal transferase-mediated DNA nick end labelling (TUNEL) assay. Apoptotic cells were identified using an in *situ cell* death detection kit, pod (Roche, Switzerland). The paraffin sections of myocardial tissue (3 μm-thickness) were deparaffinized for 5 min in xylone twice, and then rehydrated in a series of serially diluted ethanol solutions (100%, 95%, 90%, 80%, 70%, and then H_2_O). Tissue sections were then treated with proteinase K (final concentration of 20 ug/ml in 10 mM Tris–HCl, pH 7.4–7.8) for 30 min at 22°C. A positive control was prepared by incubating a tissue section with DNase I (1 U/ml in 50 mM Tris–HCl, pH7.5, 1 mg/ml BSA) for 10 min before proteinase K treatment. The paraffin sections incubated with 50 μl TUNEL reaction mixture containing terminal deoxynucleotidyl transferase (TdT) for 60 minutes at 37°C in a humidified chamber in the dark. A negative control was prepared without TUNEL enzyme. Tissue sections were washed three times with PBS and the TUNEL-positive apoptotic cardiomyocyte nuclei were examined under a fluorescence microscope using an excitation wavelength in the range of 450–550 nm and a detection wavelength in the range of 515–565 nm. Tissue sections were then treated with converter-POD in a humidified chamber for 30 min at 37 μ. and incubated with 50 ul DAB for 10 min. Positive staining cells were manually counted in 10 randomly selected fields of each slide by microscope (200× magnification field). Cell death was expressed as the average percentage of total cells counted.

### Assay of myocardial virus concentration

For the infectivity assay, portions of the heart were weighed and homogenized aseptically in 2 mL PBS. After a 15-minute centrifugation at 1500 *g*, virus titers in the supernatants were determined by a plaque assay method, as previously described [38]. In brief, VERO (African green monkey kidney) cells suspended (1 × 10^6^/mL) in Eagle’s minimal essential medium (EMEM) with 5% FCS plus 100 μg/mL each penicillin and streptomycin were placed in six-well plates and allowed to grow for 2 or 3 days at 37°C in 5% CO2. After adsorption, the cells were overlaid with 3 mL EMEM containing 5% FCS and 1% methylcellulose. After a 2-day incubation at 37°C in a humidified atmosphere containing 5% CO2, the cells were fixed with acetic acid and methanol (1:3) and stained with 1% crystal violet; plaques were then counted with an inverted microscope. The myocardial virus titer was expressed as log pfu/mg of heart.

### Western blot analysis for CREB and phosphorylated CREB

Heart tissues were homogenized and dissolved with RIPA buffer [150 mM NaCl, 1% Triton X-100, 1% sodium deoxycholate, 0.1% SDS, 50 mM Tris (pH 7.4)] containing 1 M PMSF (Phenylmethanesulfonyl fluoride). Protein concentrations were determined with the BCA Protein Assay Kit. Total protein (50 ug per lane) was separated on 12% SDS-PAGE (sodium dodecyl sulfate polyacrylamide gel electrophoresis) and transferred to a PVDF membrane (BioRad, Hercules, CA). Membranes were incubated with primary antibodies according to the manufacturer’s protocol, subsequently washed with PBS-T (phosphate-buffered saline Tween-20), and were incubated with an appropriate secondary antibody dependent to primary antibody. Results were scanned and quantified using densitometry and ImageQuant software and normalized to the housekeeping protein GAPDH. Phosphorylated CREB (pCREB), CREB and GAPDH antibodies were obtained from Cell Signaling Technology (Danvers, MA, USA). The experiment was repeated 3 times for each sample.

### Total RNA extraction and reverse transcriptase-polymerase chain reaction (RT-PCR)

Total RNA, frozen in liquid nitrogen, was extracted from the myocardial samples by the Trizol method (Invitrogen, Carlsbad, CA, USA) according to the manufacturer’s instructions. cDNA was synthesized by reverse transcription using total RNA (3 μg) as a template. Semiquantitative RT-PCR was used to detect the mRNA abundance of interleukin (IL)-6, tumor necrosis factor (TNF)-α, and monocyte chemoattractant protein-1 (MCP-1) (Table 1). Additionally, RT-PCR was also used to detect the CVB3 RNA abundance in the infected myocardium. The mRNA abundance was quantified as optical densities (OD) equalized with β-actin mRNA levels using a BandScan 5.0 software (Glyko, Novato, CA, USA). Nucleicacid sequences of all PCR products were confirmed to be identical to published GenBank data. The experiment was repeated 3 times for each sample.

**Table 1:** Primer sequences used for semi-quantitative RT-PCR

***mRNA***	***Primers***	***Annealing temperature ( ° C)***	***Cycles***	***Product (bp)***
CVB3	F-CGGTACCTTTGTGCGCCTGT	61	30	314
	R-CAGGCCGCCAACGCAGCC			
IL-6	F- TGCTGGTGACAACCACGGCC	60	33	308
	R- GTACTCCAGAAGACCAGAGG			
TNF-α	F-CCTGTAGCCCACGTCGTAGC	50	31	374
	R-TTGACCTCAGCGCTGAGTTG			
MCP-1	F-GCCAACTCTCACTGAAGCC	50	30	161
	R-GCTGGTGAATGAGTAGCAGC			
β-actin	F-AGGGAAATCGTGCGTGACAT	55	24	450
	R-CATCTGCTGGAAGGTGGACA			

### Fluorescent quantitative real-time PCR

Total RNA, frozen in liquid nitrogen, was extracted from the myocardial samples by the Trizol method (Invitrogen, Carlsbad, CA, USA) according to the manufacturer’s instructions. RNA concentration were quantified using the spectrophotometer. cDNA was synthesized by reverse transcription using total RNA (2 μg) as a template. The internal control gene used was GAPDH. For PCR amplification, the following mouse-specific sense and antisense primers were used: IL-6, 5′- TGCCTTCTTGGGACTGAT-3′ (forward) and 5′- TAAGCCTCCGACTTGTGA-3′ (reverse); TNF-а, 5′- CCACGCTCTTCTGTCTACTGA-3′ (forward) and 5′- AAGGTACAACCCATCGGCTG-3′ (reverse); GAPDH, 5′-AGGGAAATCGTGCGTGACAT-3′ (forward) and 5′- CATCTGCTGGAAGGTGGACA-3′ (reverse). PCR amplification was performed on a Light Cycler FastStart DNA Master SYBR Green I. The amplification was performed in a Roche Light Cycler 480 instrument under the following condition: initial denaturation at 95°C for 5 minutes, 50 cycles of amplification at 95°C for 10 seconds, annealing at 60°C for 10 seconds, and then extension at 72°C for 10 seconds.

### Enzyme-linked immunosorbent assay for cytokines in the heart

Cytokine levels were measured with various enzyme-linked immunosorbent assay (ELISA) kits manufactured by Westang Biotech Co Ltd (Shanghai, China) for IL-6, TNF-α, and MCP-1. The sensitivity of the kit is 16 pg/ml for IL-6, 13 pg/ml for TNF-α, and 8 pg/ml for MCP-1. Cytokine levels are expressed as pg/mg of heart. The experiment was repeated 3 times for each sample.

### Statistical analysis

All values were expressed as mean value ± standard error (SE). Survival rate was analyzed by the Kaplan-Meier method. The continuous variables were compared with a Student’s t-test for two groups and with one way analysis of variance (ANOVA) for >2 groups. Bivariate correlations were performed by Pearson’s correlation. A value of P < 0.05 was considered significant.

## Results

### Plasma noradrenaline levels

**Figure 1:**
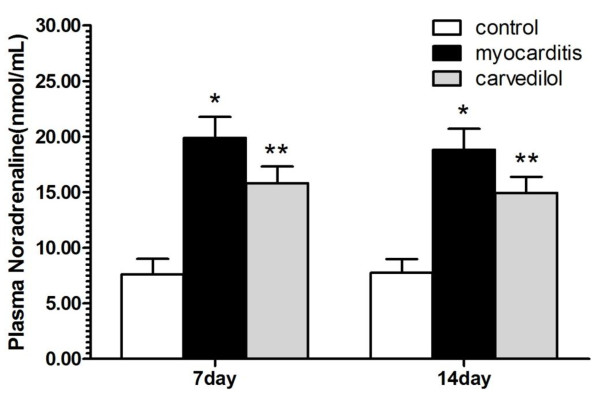
**The effects of carvedilol on plasma noradrenaline on days 7 and 14 (n = 8 in each group).** Control, normal mice treated with normal saline solution; Myocarditis, infected mice treated with normal saline solution; Carvedilol, infected mice treated with carvedilol. *P < 0.05 versus control; **P < 0.05 versus myocarditis.

Plasma noradrenaline levels were significantly higher in the carvedilol and myocarditis groups than in the normal control group on days 7 and 14 (Figure 1). However carvedilol treatment notably decreased the plasma levels of noradrenaline compared to the myocarditis group on days 7 and 14 (Figure 1).

### Hemodynamics of mice

**Figure 2:**
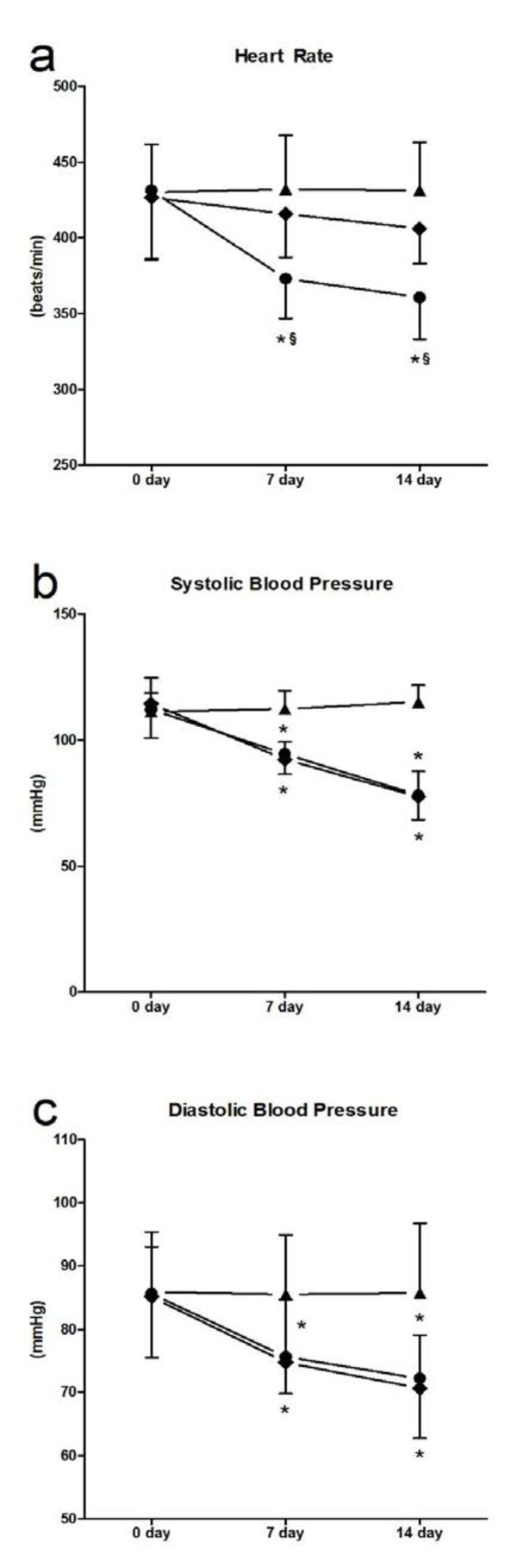
**The effects of carvedilol on heart rate and blood pressure on days 7 and 14 (n = 8 in each group). a**, heart rate; **b**, systolic blood pressure; **c**, diastolic blood pressure. ▲, Control; ◆myocarditis; ●carvedilol treatment. Control, normal mice treated with normal saline solution; Myocarditis, infected mice treated with normal saline solution; Carvedilol, infected mice treated with carvedilol. *P<0.05 versus control; §P<0.05 versus myocarditis.

HRs in the carvedilol group were significantly decreased compared with the normal and myocarditis groups on days 7 and 14 (Figure 2a). Systolic blood pressure (SBP) in the carvedilol group and myocarditis group were significantly decreased compared with the normal group on days 7 and 14 (Figure 2b). No differences in SBP and diastolic blood pressure (DBP) were found between the carvedilol and myocarditis groups on days 7 and 14 (Figure 2c).

### Survival rate

**Figure 3:**
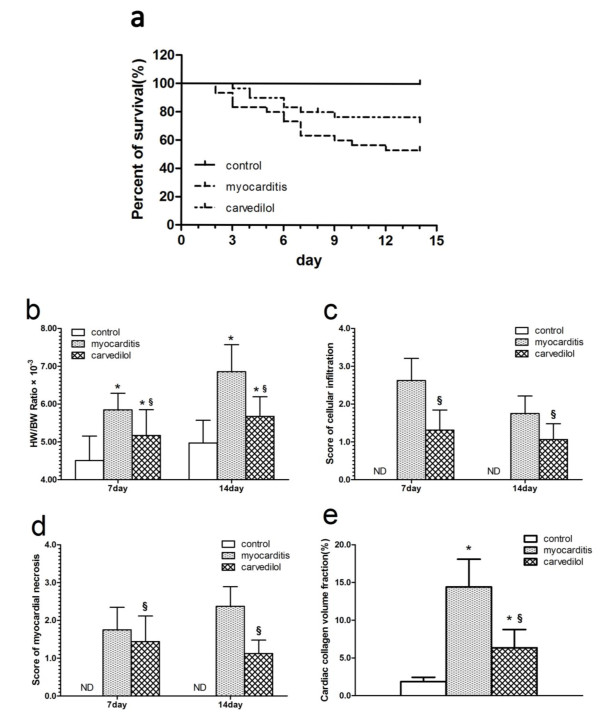
**Effects of carvedilol on viral myocarditis. a**: The survival rate in CVB3-inoculated mice followed for 14 days (n = 30 in each group). Carvedilol significantly increased the survival rate in CVB3 infected mice compared to the myocarditis group (P < 0.05). **b**: HW/BW ratio (n = 8 in each group); **c**: Histopathological score of cellular infiltration (n = 8 in each group); **d**: Histopathological score of myocardial necrosis (n = 8 in each group); **e**: Total cardiac collagen volume fraction on day 14 (n = 8 in each group). ND, not detected. Control, normal mice treated with normal saline solution; Myocarditis, infected mice treated with normal saline solution; Carvedilol, infected mice treated with carvedilol.

The survival rate of the uninfected mice (control group) on day 14 were 100% (Figure 3a). The survival rate in CVB3-inoculated mice followed for 14 days was 53.1% for those treated with saline and 73.0% for those treated with carvedilol (Figure 3a). The survival rate was significantly increased in the carvedilol group compared to the myocarditis group (P < 0.05).

### HW/BW ratio

Seven and 14 days after infection, the myocarditis group showed a reduction in BW and HW and an increase in HW/BW ratio. The HW/BW ratio was significantly lower in the carvedilol group than in the myocarditis group on days 7 and 14 (Figure 3b).

### Myocardial histopathology and fibrosis

**Figure 4:**
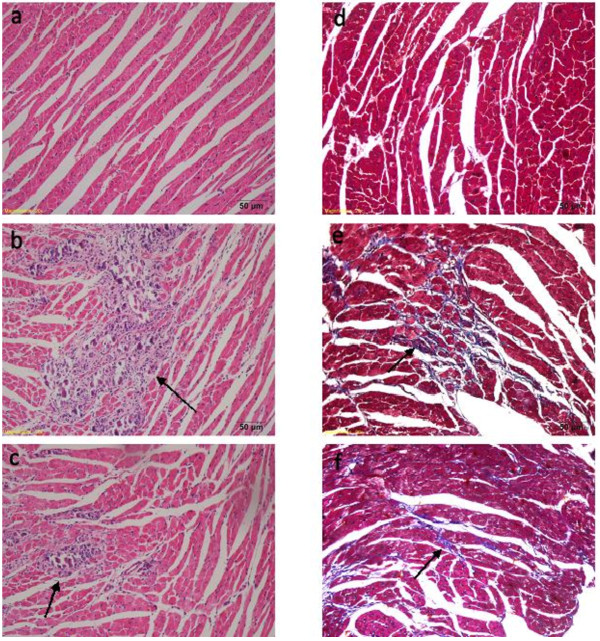
**Histopathology (Hematoxylin Eosin × 200) and fibrosis (Trichrome × 200) in the heart. (a)** Histopathology in a normal group (grade 0). **(b)** Representative histopathology in a myocarditis group. Several large foci of cellular infiltrations (arrow) in the inflammatory region are shown (grade 3). **(c)** Representative histopathology in a carvedilol group. Several small foci of cellular infiltrations in the inflammatory region (arrow) are shown (grade 2). **(d)** Representative Masson’s trichrome stained heart in a normal group. **(e)** Representative Masson’s trichrome stained heart in a myocarditis group. **(f)** Representative Masson’s trichrome stained heart in a carvedilol group.

On days 7 and 14 at sacrifice, severe injuries to myocardium with cellular infiltration in the myocarditis group were observed. The cardiac pathological scores, including infiltration, necrosis, and myocardial CVF, were all significantly decreased in the carvedilol group compared with the myocarditis group (Figure 3c,d,e), indicating a significantly reduced severity of disease. Representative hematoxylin-eosin and Masson’s trichrome stained hearts are shown in Figure 4.

### Cardiomyocyte apoptosis

**Figure 5:**
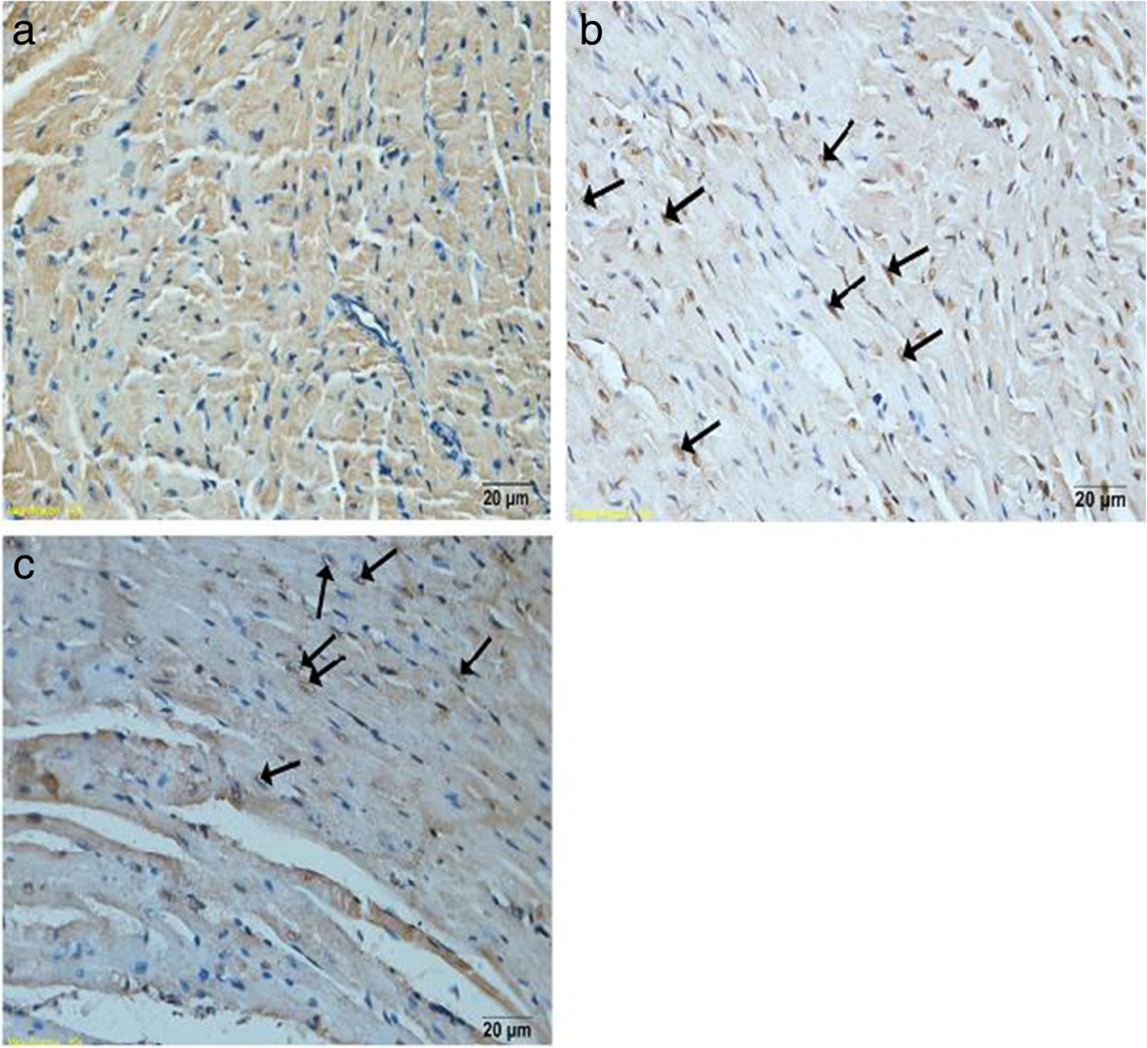
**Detection of apoptotic cardiomyocytes with the TUNEL assay (n** = **8 in each group).** Nuclei with brown staining indicated TUNEL positive cells, which were indicated by a small white arrow. **(a)** normal group. **(b)** myocarditis group. **(c)** carvedilol group.

The TUNEL positive cells in the myocardium of the infected mice were significantly increased compared with the normal mice. Carvedilol treatment attenuated the increase in percentages of apoptosis significantly (P < 0.05) compared with the myocarditis group on days 7 (5.43 ± 1.22% vs 7.29 ± 1.93%) and 14 (1.91 ± 0.72% vs 3.49 ± 1.40%). The TUNEL positive cell nuclei appeared condensed and rounded, showing typical features of apoptotic morphology (Figure 5).

### Virus titer of the heart

**Figure 6:**
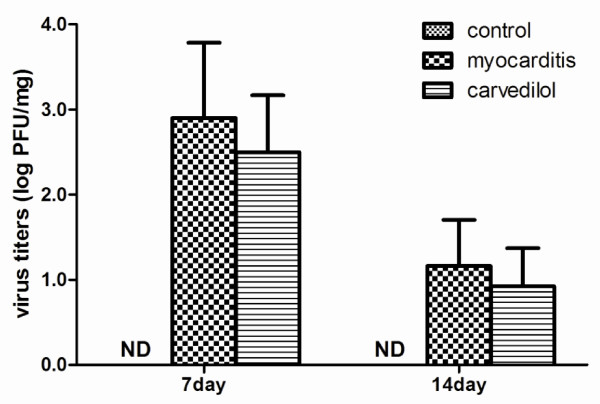
**Virus titers of hearts in the infected mice on days 7 and 14 (n** = **8 in each group).** Control, normal mice treated with normal saline solution; Myocarditis, infected mice treated with normal saline solution; Carvedilol, infected mice treated with carvedilol.

Virus titers of hearts in the infected mice were slightly lower in the carvedilol group than in the myocarditis group on days 7 and 14, but this effect did not reach statistical significance (p > 0.05, Figure 6).

### Viral genome in the myocardium

**Figure 7:**
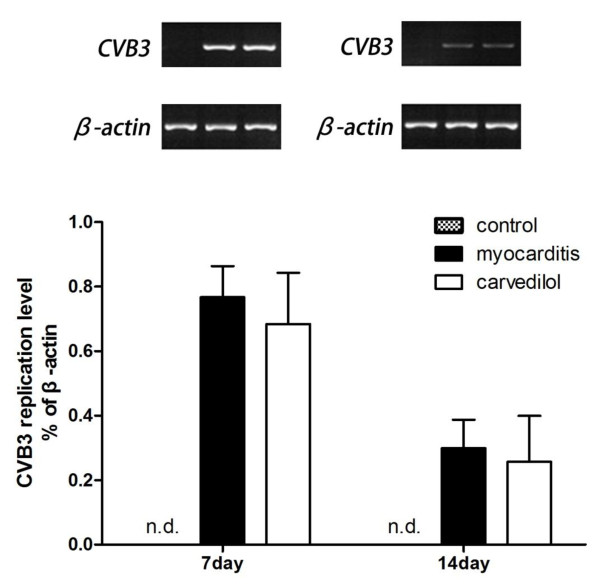
**CVB-3 RNA abundance in the infected myocardium of mice on days 7 and 14 (n** = **8 in each group).** Carvedilol slightly reduced the CVB-3 RNA abundance compared to the myocarditis group. nd, not detected. Control, normal mice treated with normal saline solution; Myocarditis, infected mice treated with normal saline solution; Carvedilol, infected mice treated with carvedilol.

CVB3-RNA abundance by semiquantitative RT-PCR-analysis were found in the myocardium of the infected mice on days 7 and 14. Carvedilol treatment produced a slightly lower CVB3-RNA abundance in the infected myocardium compared to the myocarditis group on day 7, but this effect did not reach statistical significance (Figure 7). There were no significant differences in the CVB3-RNA abundance between the carvedilol group and myocarditis group on day 14.

### Protein assay of CREB and pCREB

**Figure 8:**
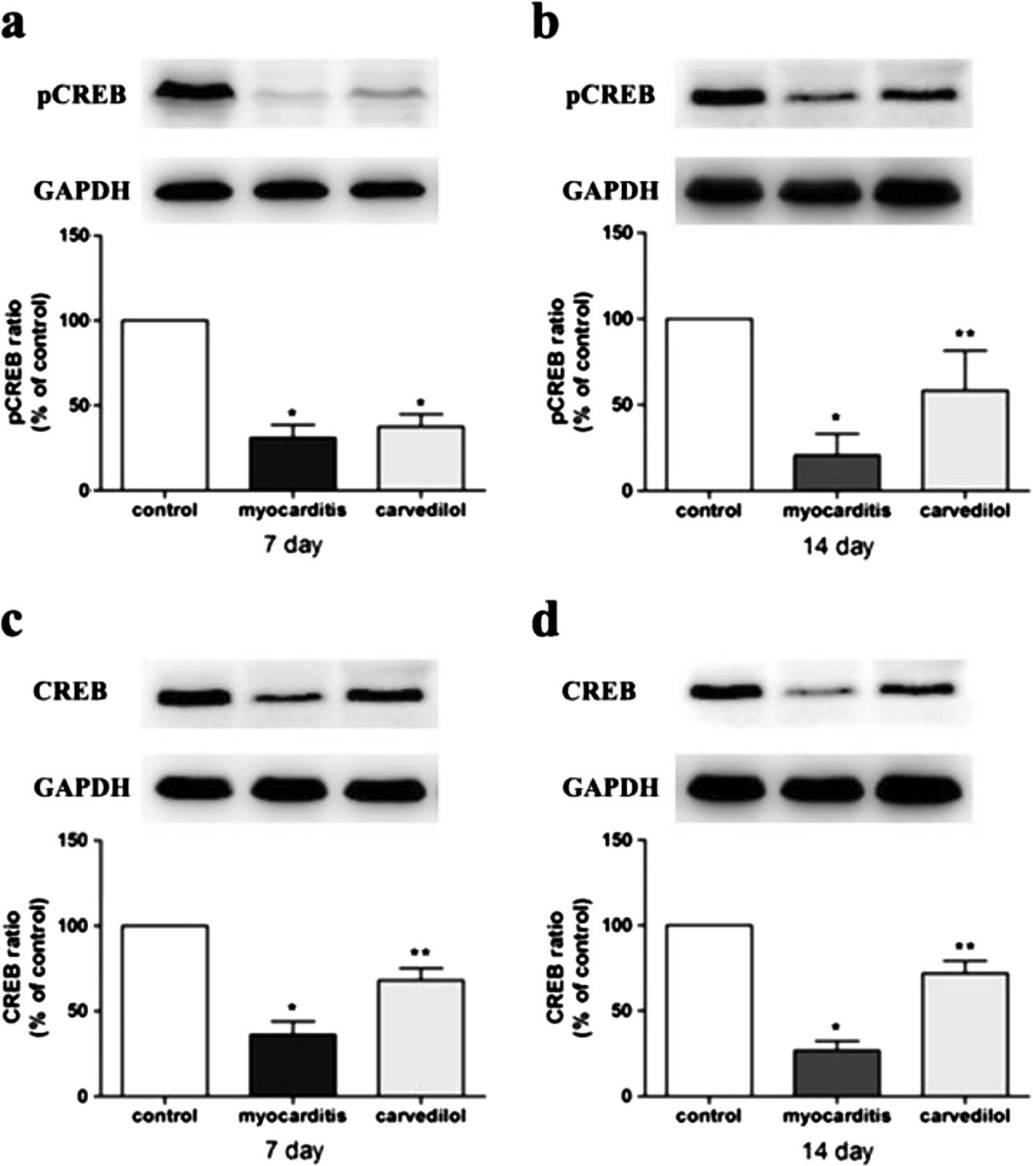
**The effects of carvedilol on cardiac phosphorylated CREB and CREB on days 7 and 14 (n = 8 in each group). a**, phosphorylated CREB (pCREB) on day 7; **b**, phosphorylated CREB (pCREB) on day 14; **c**, CREB on day 7; **d**, CREB on day 14. *P<0.05 versus control; **P<0.05 versus myocarditis. Control, normal mice treated with normal saline solution; Myocarditis, infected mice treated with normal saline solution; Carvedilol, infected mice treated with carvedilol.

In the myocardium of the infected mice western blot analysis revealed a significant decrease in the levels of CREB and pCREB compared to the uninfected animals on days 7 and 14 (Figure 8). On day 7, the levels of CREB in the carvedilol group were significantly increased compared with the myocarditis group, but the levels of pCREB of the carvedilol group did not differ compared with the myocarditis group (Figure 8). On day 14, the levels of CREB and pCREB in the carvedilol group significantly increased compared to the myocarditis group (Figure 8).

### Gene expression of cytokines by Semi-Quantitative PCR analysis in the heart

**Figure 9:**
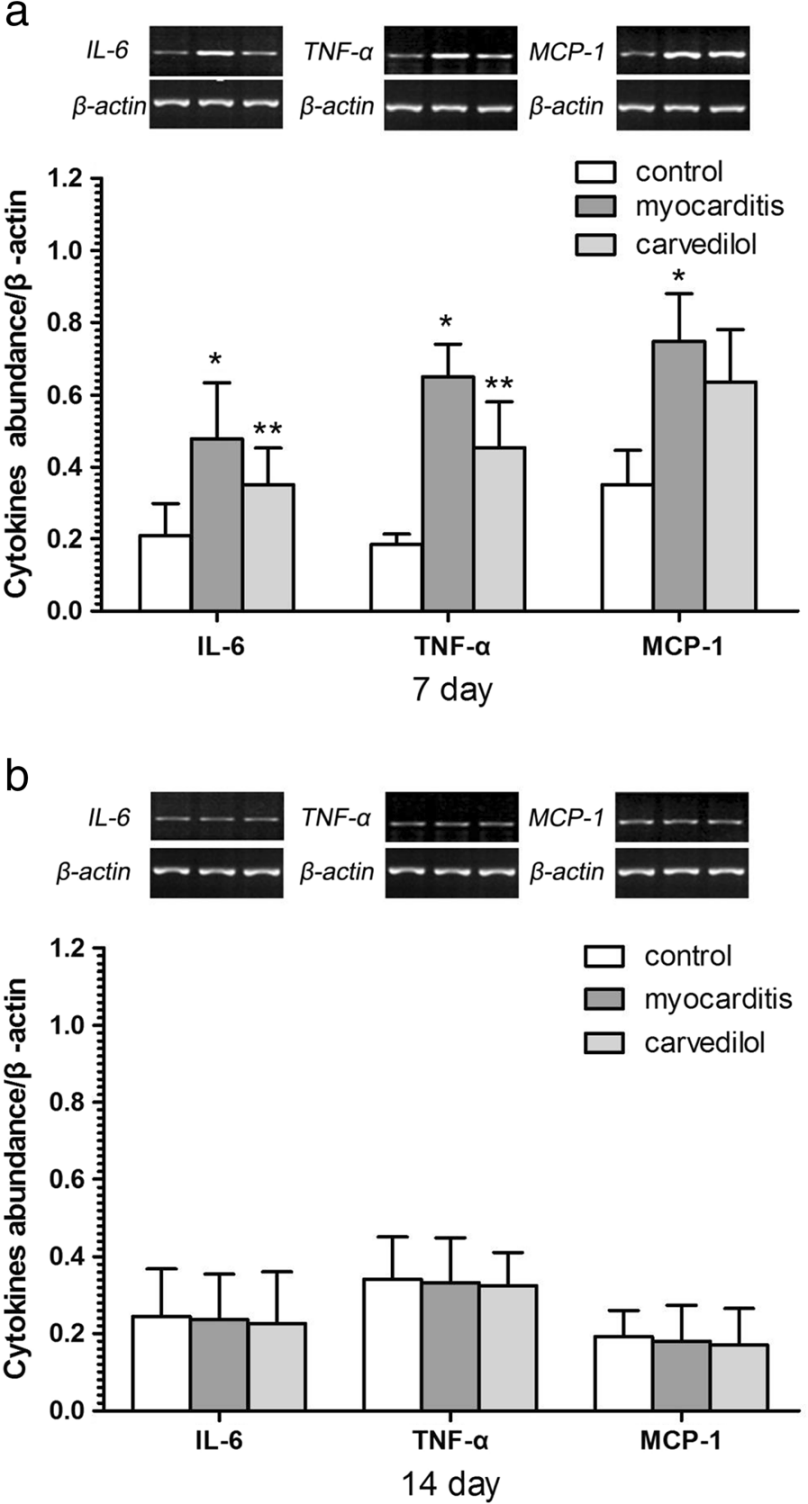
**Expression of cytokine mRNAs by Semi-Quantitative PCR analysis in the myocardial tissues of mice on days 7 and 14 (n = 8 in each group). a**, cytokine mRNAs on day 7; **b**, cytokine mRNAs on day 14. Control, normal mice treated with normal saline solution; Myocarditis, infected mice treated with normal saline solution; Carvedilol, infected mice treated with carvedilol. *P<0.05 versus control; **P<0.05 versus myocarditis.

On day 7, the mRNA levels of the IL-6, TNF-α, and MCP-1 in the myocardium of the infected mice were significantly upregulated compared with the normal group (Figure 9). On day 7, the mRNA levels of the IL-6 and TNF-α were significantly lesser in the carvedilol group compared with the myocarditis group (P < 0.05; Figure 9a), but carvedilol had no effect on the MCP-1. On day 14, no differences in the mRNA levels of the IL-6, TNF-α, and MCP-1 were found among the carvedilol group and myocarditis group and normal control group (P > 0.05; Figure 9b).

### Quantitative PCR analysis of cytokine mRNA expressions in the heart

**Figure 10:**
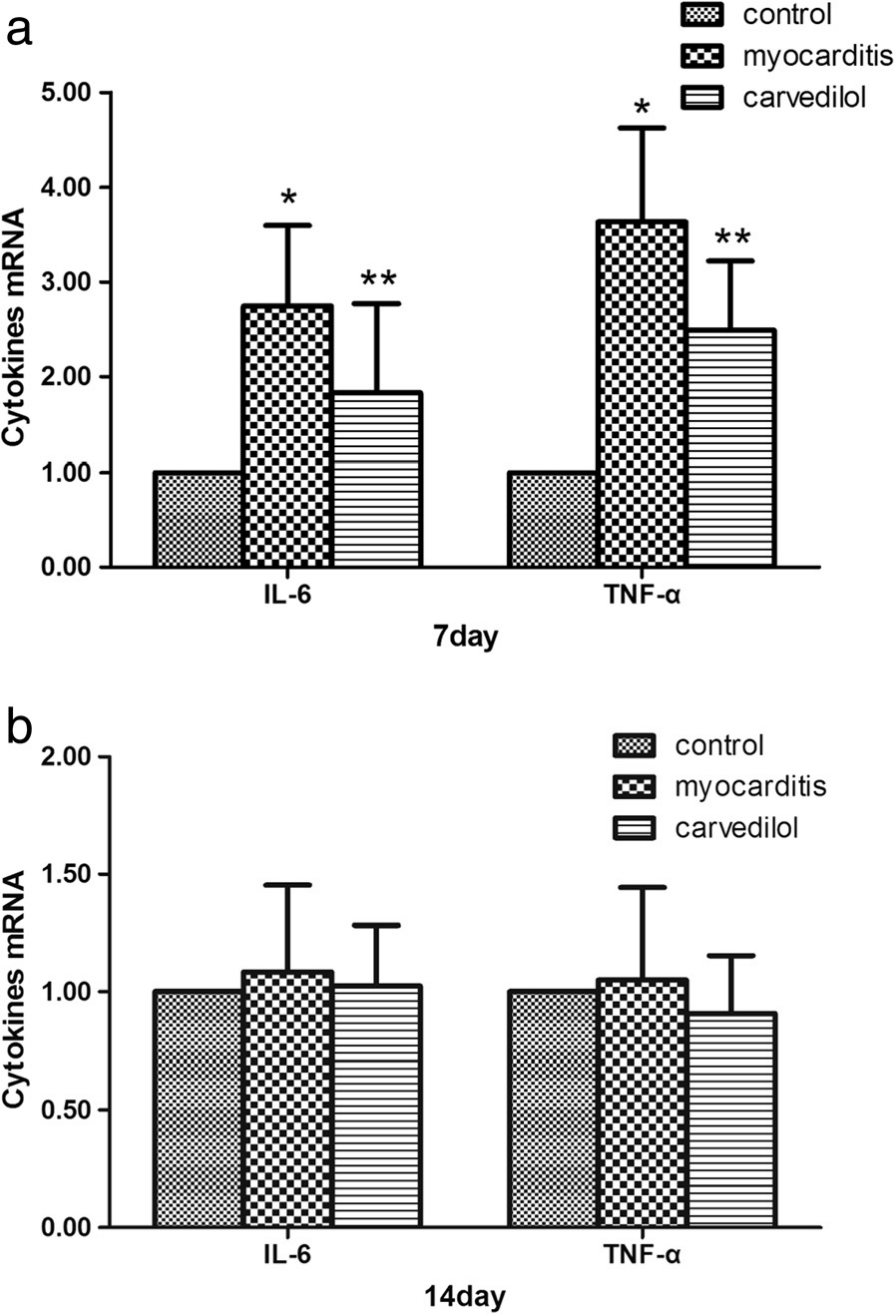
**Expression of cytokine mRNAs by Quantitative PCR analysis in the myocardial tissues of mice on days 7 and 14 (n = 8 in each group). a**, cytokine mRNAs on day 7; **b**, cytokine mRNAs on day 14. Control, normal mice treated with normal saline solution; Myocarditis, infected mice treated with normal saline solution; Carvedilol, infected mice treated with carvedilol. *P<0.05 versus control; **P<0.05 versus myocarditis.

On day 7, the mRNA levels of the IL-6 and TNF-α in the myocarditis group were significantly upregulated compared with the normal group (Figure 10). On day 7, the mRNA levels of the IL-6 and TNF-α were significantly lesser in the carvedilol group compared with the myocarditis group (P < 0.05; Figure 10a). On day 14, no differences in the mRNA levels of the IL-6 and TNF-α were found among the carvedilol group and myocarditis group and normal control group (P > 0.05; Figure 10b).

### ELISA analysis of cytokines levels in the heart

**Figure 11:**
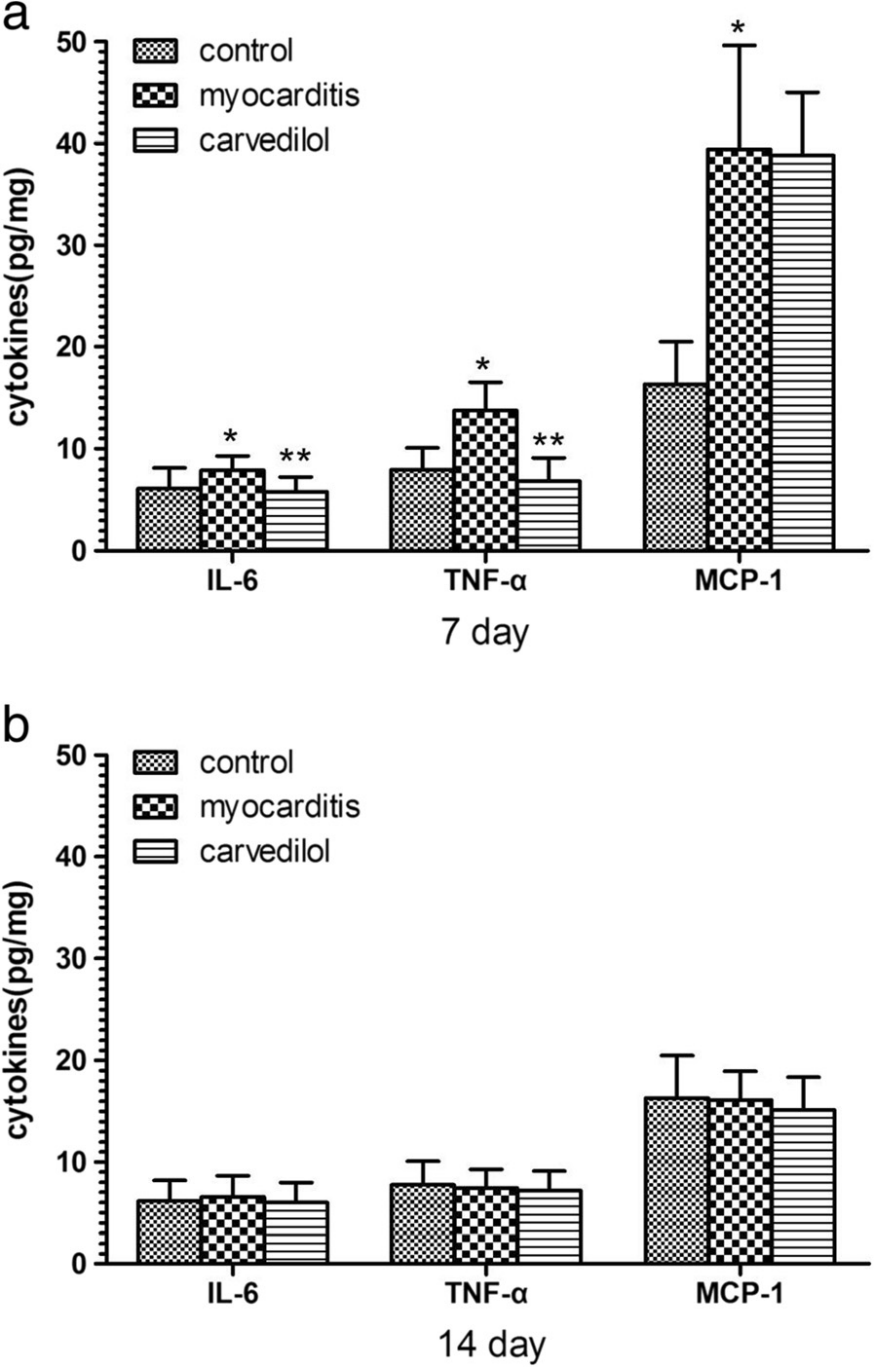
**Cytokine levels measured by ELISA analysis in the myocardial tissues of mice on days 7 and 14 (n = 8 in each group). a**, level of cytokines on day 7; **b**, level of cytokines on day 14. Control, normal mice treated with normal saline solution; Myocarditis, infected mice treated with normal saline solution; Carvedilol, infected mice treated with carvedilol. *P<0.05 versus control; **P<0.05 versus myocarditis.

On day 7, the levels of TNF-α and IL-6 were significantly lower in the carvedilol group compared with the myocarditis group (P < 0.05; Figure 11a), but carvedilol had no effect on the MCP-1. On day 14, no differences in the levels of the IL-6, TNF-α, and MCP-1 were found among the carvedilol group and myocarditis group and normal control group (P > 0.05; Figure 11b).

### Correlation between the expression of CREB, pCREB and the levels of inflammatory cytokines

**Figure 12:**
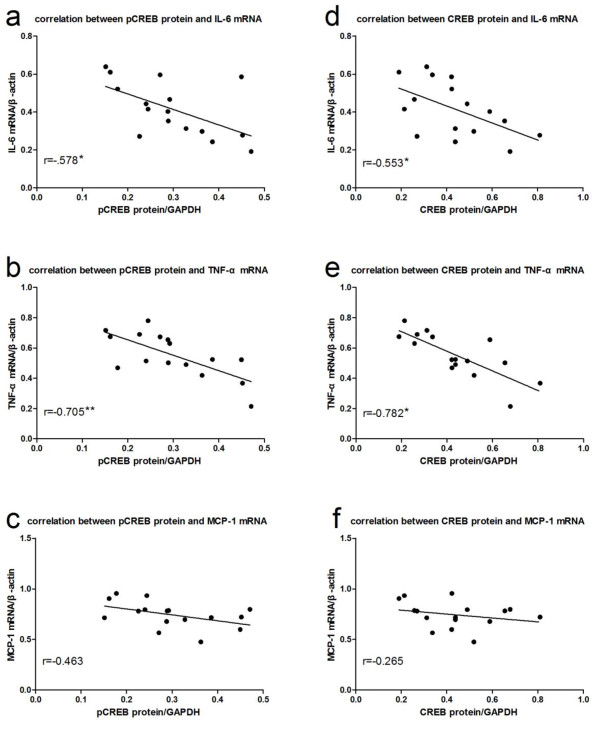
**Correlation between pCREB, CREB and inflammatory cytokines.** Pearson's correlation analysis demonstrated that the protein expression of pCREB and CREB was negatively correlated with the expression of IL-6 and TNF-α mRNA on day 7, but not correlated with the expression of MCP-1 mRNA. **a**, Correlation between pCREB protein and IL-6 mRNA; **b**, Correlation between pCREB protein and TNF-αmRNA; **c**, Correlation between pCREB protein and MCP-1 mRNA; **d**, Correlation between CREB protein and IL-6 mRNA; **e**, Correlation between CREB protein and TNF-αmRNA; **f**, Correlation between CREB protein and MCP-1 mRNA. *P<0.05; **P<0.01.

We used the Pearson’s correlation analysis method to assess the relationship between the expression of CREB or pCREB and the levels of inflammatory cytokines. Bivariate correlations analysis fit a straight line to the significant negative relationship between the expression of CREB or pCREB and the levels of IL-6 (r = −0.553 and −0.578, respectively; Figure 12) or TNF-α (r = −0.782 and −0.705, respectively; Figure 12) on day 7. The expression of CREB and pCREB was not correlated with the levels of MCP-1 mRNA (r = −0.265 and −0.463, respectively, P > 0.05).

## Discussion

Two major findings were obtained in the present study. First, the phosphorylation and expression of CREB were decreased with concomitant increase of proinflammatory cytokines in murine coxsackievirus-induced acute viral myocarditis. The levels of IL-6 and TNF-α were correlated with the expression of CREB or pCREB. This finding indicates that the cardiac transcriptional factor CREB may be involved in the pathophysiology of viral myocarditis. Second, carvedilol increased cardiac CREB expression and phosphorylation and decreased the plasma catecholamine levels and the production of IL-6 and TNF-α with amelioration of acute viral myocarditis. These results show that by blocking catecholamine stimulation and increasing the cardiac CREB expression and phosphorylation, carvedilol exerts some of its beneficial effects by suppressing the IL-6 and TNF-α. To the best of our knowledge, this is the first study to investigate the effects of carvedilol on cardiac CREB expression and phosphorylation in viral myocarditis.

### CREB expression and phosphorylation in viral myocarditis

It is well-known that stimulation of β-adrenergic receptor by catecholamines results in the elevation in the intracellular concentration of the second messenger cAMP [39]. Increased production of cAMP leads to activation of protein kinase A, which in turn causes phosphorylation/activation of CREB and subsequent gene expression through CRE-mediated transcription [20]. Recent studies have indicated an important role of CREB in the development of the cardiovascular diseases such as heart failure, atherosclerosis, restenosis, and reperfusion injury [21-29]. Moreover, emerging evidence has revealed specific functions of CREB in immune responses, including regulating NF-kB activation [40,41], inducing macrophage survival [40,42,43], and promoting the proliferation, survival, and regulation of T and B lymphocytes [40,42,43]. It has been suggested that CREB is involved in suppression of cytokine gene expression, including IL-6 and TNF-α, and promotes anti-inflammatory immune responses [18,40,41,44-48]. In the present study, CREB expression and phosphorylation were decreased with concomitant increase of IL-6 and TNF-α. The levels of IL-6 and TNF-α were correlated with the expression of CREB or pCREB. It has been suggested that cytokines exert an important role in the pathophysiology of viral myocarditis [49-53]. IL-6 and TNF-α are both involved in the pathogenesis of myocarditis and may induce advanced cardiac dysfunction [50,52,53]. CREB may thus play an important role in the pathogenesis of viral myocarditis. The reduction in CREB expression and phosphorylation in viral myocarditis may be caused by several mechanisms. First, prolonged explosure to elevated catecholamine levels lead to the desensitization of cardiac β-adrenergic receptors signaling that limit cAMP production and reduce the expression and phosphorylation of CREB [11,54]. Previous studies have demonstrated that cardiac CREB alteration was associated with catecholamines stimulation time.^22,54^ CREB mRNA was increased after short-term (30 min) stimulation with forskolin, an activator of cAMP [55], while prolonged β-adrenergic stimulation in rats by a 4-day infusion of isoproterenol decreased CREB mRNA [22]. Second, a direct proteolytic cleavage of CREB by viruses may lead to the reduction in CREB. A few studies have demonstrated that infection of susceptible cells with members of the picornavirus family results in the rapid inhibition of host cell RNA synthesis [56-59]. Yalamanchili et al. found that CREB and pCREB were specifically cleaved by the poliovirus-encoded protease 3C ^pro^ both in vitro and in virus-infected cells [60]. Therefore, CVB3 may directly cleave the CREB protein and result in the reduction of CREB.

### Effects of Carvedilol on CREB expression and phosphorylation in viral myocarditis

In this study, treatment with carvedilol decreased the plasma levels of noradrenaline and increased the expression and phosphorylation of cardiac CREB. As mentioned previously [11,54], elevated circulating catecholamines may lead to decreased levels and functional activity of cardiac β-adrenergic receptors and thus to marked desensitization of the heart to inotropic β-adrenergic stimulation. Treatment with carvedilol may normalize or remodel signaling through the cardiac β-adrenergic system by reducing desensitization, enhancing catecholamine sensitivity, and raising levels of β-adrenergic receptors [11,32,54]. Thus, decreased β-adrenergic stimulation by carvedilol treatment may contribute to the increase in the expression and phosphorylation of CREB. In agreement with previous studies from our and other laboratories [30,31,33-35], we found that carvedilol improved the survival of mice and reduced myocardial inflammation and necrosis in murine viral myocarditis by downregulating the production of IL-6 and TNF-α. In this study, we found that the levels of IL-6 and TNF-α was negatively correlated with the cardiac CREB and pCREB. Moreover, previous studies have found that CREB was involved in suppression of IL-6 and TNF-α gene expression in other systems [18,40,41,46-48]. Therefore, the reduction in the production of IL-6 and TNF-α may be associated with the increase of CREB in the study.

Recent reports have also emphasized the other cellular mechanisms in murine myocarditis [61-65]. After viral entry acute injury of the myocytes induced by virus replication leads to myocyte necrosis, exposure of intracellular antigens (e.g., cardiacmyosin), and activation of the host’s immune system, which is characterized by the invasion of natural killer cells and macrophages followed by T lymphocytes. After the acute phase of virus-induced injury, the second phase is characterized by (auto)immune reactions. This subacute phase is defined by activated virus-specificT lymphocytes, which may target the host’s organs by molecular mimicry. Kania and Shi et al. reported that toll-like receptors transmit a cascade of signals to activate nuclear transcription factors, such as nuclear factor κB, and lead to inflammatory cytokine production and immune activation. Cytokine activation (IL-6 and TNF-α) and antibodies to viral and cardiac proteins may aggravate cardiac damage and cause impairment of the contractile function.

### Study limitations

The survival rate, HW/BW ratio and myocardial histopathology of infected mice were studied in the present study. However, cardiac function and volume were not studied by echocardiographic examination. In addition, although the cardiac transcriptional factor CREB appears to be involved in the pathophysiology of viral myocarditis, further research to reveal signaling pathways or collaborating proteins that are selectively involved in its proinflammatory or its anti-inflammatory functions is needed.

## Conclusions

The expression and phosphorylation of CREB were decreased in murine coxsackievirus-induced acute viral myocarditis. Administration of carvedilol causes an increase in the expression and phosphorylation of CREB together with the reduction in the production of IL-6 and TNF-α. The levels of IL-6 and TNF-α were correlated with the expression of CREB or pCREB. Although the cardiac transcriptional factor CREB appears to be involved in the pathophysiology of viral myocarditis, further research to reveal signaling pathways or collaborating proteins that are selectively involved in its proinflammatory or its anti-inflammatory functions is needed.

## Competing interests

The authors declare that they have no competing interests.

## Author’contributions

LYC designed the whole study, LYC, GLS, CYH, ZND and ZT performed the experiment, LYC and GLS wrote the paper. All authors read and approved the final manuscript.

## Pre-publication history

The pre-publication history for this paper can be accessed here:

http://www.biomedcentral.com/1471-2261/13/100/prepub
